# Ketone- and Cyano-Selenoesters to Overcome Efflux Pump, Quorum-Sensing, and Biofilm-Mediated Resistance

**DOI:** 10.3390/antibiotics9120896

**Published:** 2020-12-11

**Authors:** Nikoletta Szemerédi, Annamária Kincses, Katerina Rehorova, Lan Hoang, Noemi Salardón-Jiménez, Clotilde Sevilla-Hernández, Jitka Viktorová, Enrique Domínguez-Álvarez, Gabriella Spengler

**Affiliations:** 1 Department of Medical Microbiology and Immunobiology, Faculty of Medicine, University of Szeged, Dóm tér 10, 6720 Szeged, Hungary; szemeredi.nikoletta@med.u-szeged.hu (N.S.); kincses.annamaria90@gmail.com (A.K.); 2 Department of Biochemistry and Microbiology, Faculty of Food and Biochemical Technology, University of Chemistry and Technology Prague, Technická 3, 166 28 Prague, Czech Republic; katerina.rehorova@vscht.cz (K.R.); hoangl@vscht.cz (L.H.); jitka.prokesova@vscht.cz (J.V.); 3 Instituto de Química Orgánica General (IQOG-CSIC), Consejo Superior de Investigaciones Científicas, Juan de la Cierva 3, 28006 Madrid, Spain; noemi.sj.95@gmail.com (N.S.-J.); clo.sh.1995@gmail.com (C.S.-H.)

**Keywords:** selenoesters, *Salmonella* species, *Staphylococcus aureus*, *Pseudomonas aeruginosa*, biofilm, quorum sensing, multidrug resistance, antibacterial activity

## Abstract

The emergence of drug-resistant pathogens leads to a gradual decline in the efficacy of many antibacterial agents, which poses a serious problem for proper therapy. Multidrug resistance (MDR) mechanisms allow resistant bacteria to have limited uptake of drugs, modification of their target molecules, drug inactivation, or release of the drug into the extracellular space by efflux pumps (EPs). In previous studies, selenoesters have proved to be promising derivatives with a noteworthy antimicrobial activity. On the basis of these results, two series of novel selenoesters were synthesized to achieve more potent antibacterial activity on Gram-positive and Gram-negative bacteria. Fifteen selenoesters (eight ketone-selenoesters and seven cyano-selenoesters) were investigated with regards to their efflux pump-inhibiting, anti-quorum-sensing (QS), and anti-biofilm effects in vitro. According to the results of the antibacterial activity, the ketone-selenoesters proved to be more potent antibacterial compounds than the cyano-selenoesters. With regard to efflux pump inhibition, one cyano-selenoester on methicillin-resistant *S. aureus* and one ketone-selenoester on *Salmonella* Typhimurium were potent inhibitors. The biofilm inhibitory capacity and the ability of the derivatives to disrupt mature biofilms were noteworthy in all the experimental systems applied. Regarding QS inhibition, four ketone-selenoesters and three cyano-selenoesters exerted a noteworthy effect on *Vibrio campbellii* strains.

## 1. Introduction

The rapid emergence of multidrug-resistant bacteria is jeopardizing the effectiveness of antibiotics that have saved millions of lives previously [1]. Microbes have become resistant to common antibiotics due to the irresponsible use of the antibiotics; therefore, the appearance of resistant bacterial strains makes the treatment of infections more complicated [2]. The improper use of antibiotics has also occurred in veterinary practice and in food-producing animal farms [3]. This has led to the emergence of superbugs that are resistant to several classes of antibiotics, such as carbapenem resistant *Enterobacteriaceae* [4] and biofilm-producing methicillin-resistant *Staphylococcus aureus* (MRSA) [5].

Numerous bacterial isolates produce biofilms, which are the surface-attached bacterial cells embedded into an extracellular matrix that can protect the bacterial population against antibiotics. These biofilm-producing bacteria are more resistant to antibiotics compared to the planktonic cells, which are more susceptible to biocides [6].

It was believed that bacteria are independent and unicellular organisms [7]. Nevertheless, planktonic growth of bacteria seldom exists in nature. It has been shown that bacteria in nature exist in a large, contiguous, and dynamic surface-associated community, called biofilm, and this population has a unique behavior, namely, the properties of the community depend on population density [8]. The cells in biofilms are in contact with each other. Bacteria in the biofilm secrete small extracellular molecules to communicate with each other [9]. Several bacteria have been shown to regulate different physiological processes and activities *via* a mechanism called quorum sensing (QS), in which bacterial cells produce, detect, and reply to small diffusible signal molecules [10]. It is known that these bacteria need to achieve a critical cell density before they express virulence factors and attack the host organism.

In addition, the over-expression of bacterial efflux pumps can also contribute to bacterial multidrug resistance (MDR). The efflux pumps are transmembrane transport proteins involved in the extrusion of toxic substances into the external milieu. Furthermore, these efflux pumps might be involved in the regulation of the expression of QS-dependent virulence factors. Therefore, the inhibition of efflux pumps may decrease the virulence of resistant bacteria [11].

Different selenocompounds and selenium nanoparticles have shown a significant antibacterial and anti-biofilm activity. Among the selenoparticles (SeNPs), SeNPs synthesized using aqueous berry extract of *Murraya koenigii* showed antibacterial activity against *Enterococcus faecalis*, *Streptococcus mutans*, *Shigella sonnei*, and *Pseudomonas aeruginosa*, as well as anti-biofilm activity against *P. aeruginosa* [12]. Alternatively, SeNPs conjugated with antibiotics were potent antibacterial agents and biofilm disruptors against MRSA [13]. Among selenocompounds, a series of steroidal β-hydroxy-phenylselenides also showed antibacterial activity against *P. aeruginosa*, and prevented its biofilm formation [14]. Similarly, ebselen derivatives were anti-biofilm and potent antibacterial agents against MRSA, with minimum inhibitory concentration (MIC) values below 2 μg/mL [15].

In this context, our group reported previously that selenoesters and selenoanhydrides are bioactive selenium-containing compounds initially designed as potential anticancer and MDR-reversing agents [16] with antioxidant activity [17]. Selenium (Se) and the Se-containing compounds are known antioxidants because this essential trace element allows the antioxidant activity of the glutathione peroxidase, the enzyme that empowers the deactivation of hydrogen peroxides [18,19,20]. In line with this, patients with bacterial and viral infections generally show high oxidative stress levels, as well as low levels of selenium in blood. Besides the reduction of this oxidative stress, Se can also boost the response of the immune system against infectious diseases [21,22]. The antibacterial activity of the abovementioned selenoesters and selenoanhydrides was evaluated, finding that they showed a potent antibacterial activity against MRSA, *Salmonella enterica* serovar Typhimurium, and *Chlamydia trachomatis* serovar D. Additionally, they exerted a noteworthy anti-biofilm activity, as well as being inhibitors of bacterial efflux pumps [23,24,25].

On the basis of these antecedents, we tested the antibacterial, anti-biofilm, and anti-quorum sensing activity of 15 selenoesters in this study, comprising 8 ketoneselenoesters (R=COCH3, compounds **K1**–**K8**, Table 1) and 7 cyanoselenoesters (R=CN, compounds **N1**–**N7**, Table 1). With our selenocompounds, we aimed to reduce the intercellular communication and thus reduce biofilm formation and reverse resistance.

## 2. Results

### 2.1. Determination of Minimum Inhibitory Concentrations by Microdilution Method

On the basis of the results obtained on Gram-positive and Gram-negative bacteria, we found that the ketone-selenoesters demonstrated a strong antibacterial activity against the Gram-positive strains investigated. The most potent derivatives were **K1**, **K7**, and **K8**—they were effective on all three *S. aureus* strains tested, even reaching the submicromolar range (MIC between 0.39 and 1.56 µM, Table 2).

Regarding the cyano-selenoesters, they were also more active on Gram-positive strains. Nevertheless, they were less effective on the MRSA strains (MIC: 25*–*100 µM) compared to the methicillin-susceptible reference American Type Culture Collection (ATCC) 25923 strain (MIC: 12.5 µM). Considering this antibacterial potency, three out of the seven cyano-selenoesters evaluated seemed to be more powerful, namely, **N3**, **N6,** and **N7**. On the contrary, the ketone-selenoesters and the cyano-selenoesters were not effective on the tested *P. aeruginosa* strain and had a mild antibacterial activity on the *S.* Typhimurium strains investigated (MIC: 50*–*100 µM) (Table 2).

### 2.2. Real-Time Ethidium Bromide Accumulation Assay

**Figure 1:**
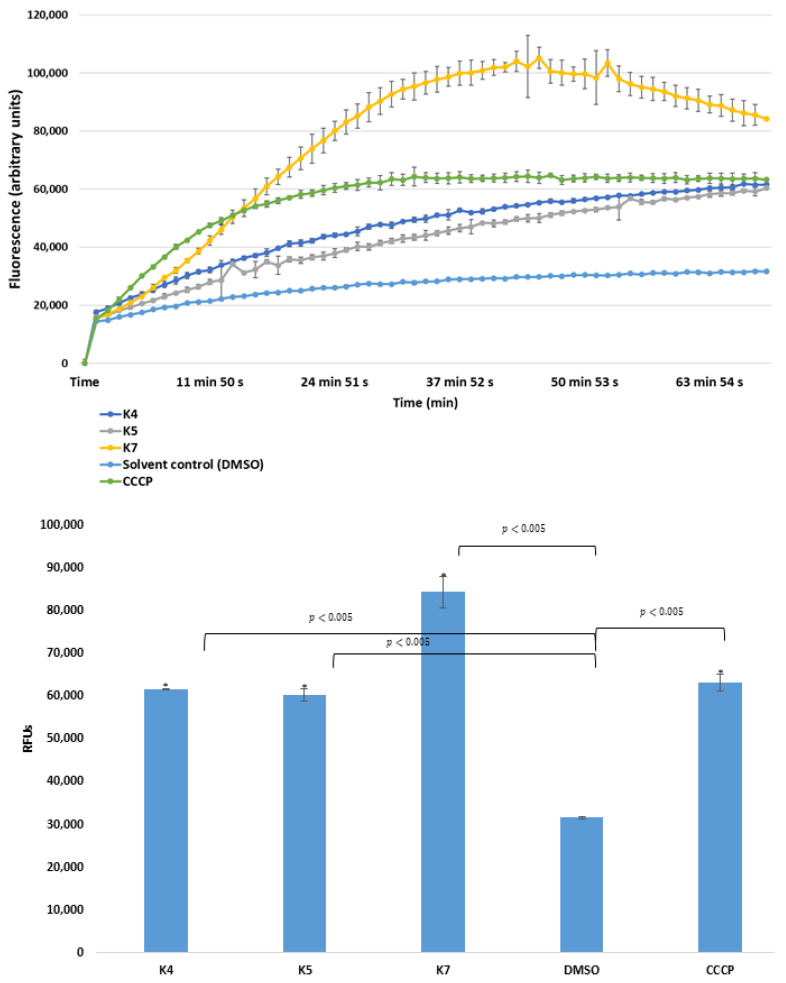
Accumulation of ethidium bromide (EB) in the presence of selenoesters **K4, K5**, and **K7** on *S. Typhimurium* SE39 Δ*tolC* strain. DMSO: dimethyl sulfoxide (solvent); CCCP: carbonyl cyanide 3-chlorophenylhydrazone (positive control). The level of significance was lower than *p* = 0.005 in all cases; α = 0.05; *p* values less than 0.005 are marked with an asterisk.

**Figure 2:**
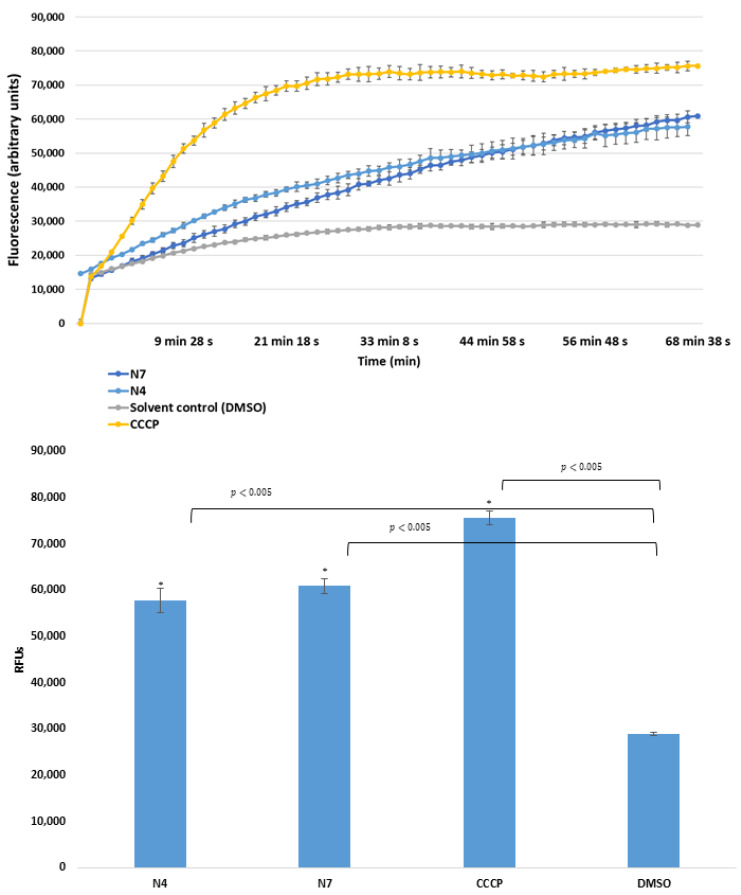
Accumulation of EB in the presence of **N4** and **N7** at one-half minimum inhibitory concentration (MIC) on *S. Typhimurium* SE39 Δ*tolC* strain. DMSO: dimethyl sulfoxide (solvent); CCCP: carbonyl cyanide 3-chlorophenylhydrazone (positive control). The level of significance was lower than *p* = 0.005 in all cases; α = 0.05; *p* values less than 0.005 are marked with an asterisk.

In this study, the ketone- and cyano-selenoesters were tested for their ability to inhibit efflux pumps on Gram-negative and Gram-positive model bacterial strains (Table 3). The efflux pump inhibitor (EPI) activity was investigated on *S. aureus* ATCC MRSA 43300 and *S.* Typhimurium SE01, SE02, SE03, and SE39 strains. Regarding the *Salmonella* strains tested, the ketone-selenoester **K7** was the most potent EPI because it increased the ethidium bromide (EB) accumulation in the efflux pump gene-inactivated mutant *S.* Typhimurium (Δ*acrA* and Δ*tolC*) strains (relative fluorescence index (RFI): 1.15 and 1.67, respectively), probably because this compound may cause membrane destabilizing effects. It was observed that **K7** inhibited the efflux activity of the wild-type SE01 strain as well (RFI: 1.02). The inhibition by **K7** in Δ*tolC* strain was stronger than inhibition in the presence of the reference compound CCCP (carbonyl cyanide m-chlorophenyl hydrazone). In addition, ketone-selenoesters **K4** and **K5** inhibited the EB accumulation in the *tolC*-inactivated mutant strain (Figure 1). Regarding cyano-selenoesters, the most pronounced activity was exerted by **N4** and **N7** on the *tolC*-inactivated mutant strain (Figure 2). The significance level was determined between the negative and positive controls and between the tested substances and the negative control. 

**Figure 3:**
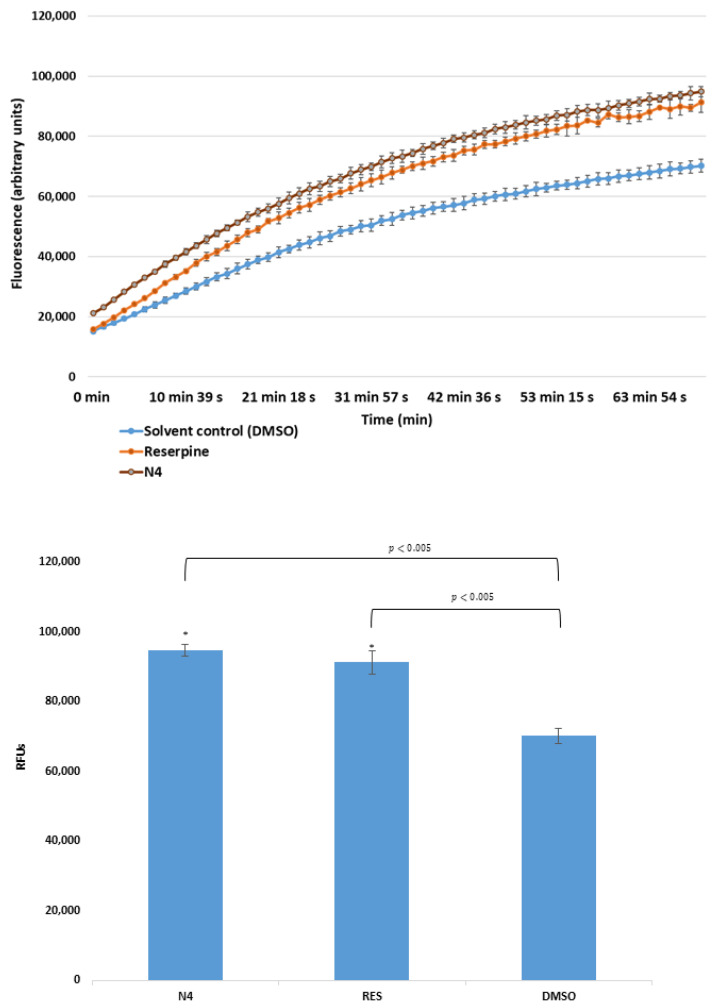
Accumulation of EB in the presence of selenoester **N4** at one-half MIC on *S. aureus* MRSA (methicillin-resistant *Staphylococcus aureus*) 43300. DMSO: dimethyl sulfoxide (solvent); reserpine: positive control. The level of significance was lower than *p* = 0.005 in all cases; α = 0.05; *p* values less than 0.005 are marked with an asterisk.

In case of *S. aureus* ATCC MRSA 43300, only one derivative (the cyano-selenoester **N4**) showed a potent EPI activity (Figure 3); in addition, this effect was more pronounced (RFI: 0.351) than the one obtained in the presence of the reference EPI reserpine (RFI: 0.300).

### 2.3. Assay for Quorum Sensing (QS) Inhibition

In this case, the concentration that halves the viability (IC_50_) was compared to the concentration halving the cell-to-cell communication (EC_50_). This was a necessary step to differentiate between the toxic concentration and the quorum-sensing inhibiting concentration. If the dose for toxicity was higher than the dose needed for quorum sensing (QS) inhibition, the tested compound was considered efficient. Therefore, the comparison of toxicity and QS inhibiting concentrations was evaluated by means of the selectivity index (SI), which was calculated as the ratio of IC_50_ and EC_50_. A higher index is related to a more potent efficacy of the compound in QS inhibition. As can be seen in Table 4, all tested compounds (except for the compound **N5**) were able to inhibit the bacterial communication.

Usually, for the practical application, indexes should be higher than 10 [26]. On the basis of this criterion, the promising ketone-selenoesters are **K1**, **K2**, and **K8**, whereas the most effective cyano-selenoester is **N2**. The ability of selenocompounds to inhibit quorum sensing was tested using two strains of *Vibrio campbellii*. The wild-type of these bacteria uses both autoinducer-1 (AI-1) and autoinducer-2 (AI-2) types of molecules for its communication. Strain 1118 is deficient in communication on the basis of AI-2, while strain 1119 is deficient in AI-1 type communication. Out of the tested compounds, only **K2** was able to inhibit the communication on the basis of either AI-1 or AI-2 molecules, with a selectivity index higher than 10 (17.5 and 14.7, respectively). The ketone-selenoester **K1** resulted in being the most promising compound in the inhibition of AI-1-based communication showing the SI of 26.2. The second most potent AI-1 inhibitor was **N2** (SI = 21.6), which was also the only cyano-selenoester capable of inhibiting AI-1-based communication. In contrast, the cyano-selenocompounds were more effective inhibitors of AI-2-based communication, with **N3** (SI = 37.6) and **N7** (SI = 30.8) being the most effective compounds among them (Table 4).

### 2.4. Anti-Biofilm Activity

The anti-biofilm activity was evaluated against typical pathogenic bacteria known for biofilm formation, such as the Gram-positive *S. aureus* and the Gram-negative *P. aeruginosa*. The ability of the compounds to affect the biofilm formation (the inhibition of cell adhesion) was tested, followed by the determination of their ability to disrupt mature biofilms. As can be seen in Table 5, all of the tested compounds were able to affect both stages of the biofilm formation. As is known, biofilm is a layer of cells protected from the adverse external conditions; therefore, the concentrations needed to halve the mature biofilm are several times higher than those needed for halving the adhesion of bacteria. This difference was most pronounced for compound **N3**, which required up to 26- and 11-fold higher concentration for achieving the disruption of the biofilm produced by *S. aureus* and *P. aeruginosa*, respectively, compared to the concentrations at which the inhibition of the adhesion takes place. In contrast, compound **K5** possessed the least noticeable difference in cell adhesion and biofilm disruption, which was only five and four times higher for *S. aureus* and *P. aeruginosa*, respectively. Almost in all cases, the selenocompounds evaluated were slightly more active against *P. aeruginosa* than against *S. aureus.*

## 3. Discussion

### 3.1. Antibacterial Activity

Previously, it was described by our group that a methylketone selenoester had antibacterial activity against Gram-positive bacteria, and two selenocompounds (a selenoanhydride and a diselenodiester) were active inhibitors of the AcrAB-TolC system [25]. In addition, a series of symmetrical selenoesters were investigated with respect to their anti-biofilm and efflux pump-inhibiting properties. In this study, we observed that the methyloxycarbonyl selenoesters showed a significant biofilm and efflux pump inhibition, and that a strong QS inhibiting activity was exerted by a methyloxycarbonyl selenoester [24].

As a continuation of our former studies, we synthesized new classes of Se-containing compounds and investigated them as potential antibacterial agents in this work. According to the results of the antibacterial activity, the ketone-selenoesters proved to be more potent antibacterial compounds than the cyano-selenoesters against the strains of *Staphylococcus aureus* evaluated. The ketone-selenoesters exerted potent activity on sensitive and methicillin-resistant *S. aureus* strains. Interestingly, the cyano-selenoesters were slightly more active than the ketone-selenoesters against the *Salmonella enterica* serovar Typhimurium strains evaluated—the seven cyano-selenoesters tested showed MIC values of 50 or 100 μM, whereas half of the eight ketone-containing selenoesters had MIC values above 100 μM. None of the 15 derivatives had antibacterial activity on *Pseudomonas aeruginosa*.

A few structure–activity relationships (SAR) can be concluded on the basis of the activity of ketone-selenoesters against *S. aureus*, taking into account that the number of compounds was not enough and thus more experiments should be performed in the future in order to confirm these empirical observations. The most active compound was **K6**, which had a *tert*-butyl group in *para*-position, with MIC values of 1.56 μM on the sensitive strains and 0.39–3.13 μM for the MRSA strains. **K6** was the unique compound with an electron-donating substituent in this work, as previous evaluations of selenoesters pointed out that electron-withdrawing substituents generally showed higher biological activity. Further studies should explore additional compounds with electron-donating substituents to confirm if they have higher antibacterial activity against *S. aureus*. In any case, the differences were small, as **K1** (unsubstituted), **K6** (3-chloro-4-fluoro substituted), and **K8** (2,4,5-trifluoro substituted) showed similar MIC values on two *S. aureus* bacterial strains.

Among the nitrile derivatives **N1**–**N7**, all exerted similar activity (MIC = 12.5 μM) against the sensitive *S. aureus* ATCC 25923 strain. Taking together the results on the MRSA and *S.* Typhimurium strains, the most active ones were **N1** (unsubstituted), **N3** (4-Br-substituted), **N6** (3-Cl-substituted), and **N7** (3,5-bis(trifluoromethyl)-substituted). These data suggest that, among monosubstituted compounds, those that include a bromine or a chlorine atom bound to the ring have better activity than those with a fluoro or a trifluoromethyl group, and that the inclusion of a second trifluoromethyl group contributes to an antibacterial activity similar to the one observed for the bromine or chlorine derivatives.

It is noteworthy to mention that the less active compound (**K4**) had a bulky substituent (trifluoromethyl group) at the *ortho* position of the selenoester. This fact may produce a steric hindrance that may hamper the hydrolysis of the selenoester inside the cells, which is the suggested mechanism underlying the biological activity [16]. When this bulky substituent was replaced by the smallest possible substituent (–H in compound **K1**), the MIC value was twofold lower on the three strains of *S. aureus* tested. Additionally, its replacement by a fluorine atom (intermediate between –H and –CF_3_) led to a twofold MIC reduction on *S. aureus* ATCC 25923 and in *S. aureus* MRSA 272123, but maintaining the MIC value on the third strain (*S. aureus* MRSA 43300). In this case, the inclusion of additional –F atoms at positions -4 and -5 (selenoester **K8**) managed to reduce the MIC value on the third strain, achieving an activity comparable to **K1**. Interestingly, the same effect of the steric hindrance was observed in the cyano-selenoesters between the compounds with a 2-CF_3_ (**N4**) and a 2-H (**N1**); as in the ketone derivatives, the bulky derivative was less active than the unsubstituted derivative.

### 3.2. Efflux Pump Inhibitory Asssay

Multidrug resistance due to drug efflux mechanisms protects bacteria through the extrusion of antibiotics out of the bacterial cells. Thus, this efflux-related phenomenon can make bacterial infections untreatable due to the lack of activity of the antibiotics. Thus, a promising strategy to restore the sensitivity of bacteria to antibiotics could be their administration together with efflux pump inhibitors, also known as EPIs [27].

In order to reverse the multidrug-resistant phenotype and re-sensitize multidrug-resistant bacteria to antibiotic therapy, the application of EPIs is an adequate approach, and natural and synthetic molecules have been described as EPIs against Gram-negative and Gram-positive bacteria [27]. Regarding the present ketone- and cyano-selenoesters, only one cyano-selenoester—**N4**—showed a potent EPI activity on methicillin-resistant *S. aureus*; furthermore, this inhibition was stronger than the effect of the reference EPI reserpine. In addition, ketone-selenoester **K7** was an effective EPI on *Salmonella* Typhimurium strains, supposedly due to its membrane-destabilizing activity. Interestingly, all of the compounds that have at least one trifluoromethyl group (**K4**, **K5**, **N4**, and **N7**), with the exception of **N5**, showed moderate efflux pump inhibitory effects on *S.* Typhimurium SE39 Δ*tolC* strain in terms of the real-time ethidium bromide accumulation assay. Consequently, this –CF_3_ moiety and the –C(CH_3_)_3_ moiety of **K7** seemed to be relevant for this efflux pump inhibition activity in *S.* Typhimurium SE39 Δ*tolC* strain.

In this work, we explored the ability of the compounds to inhibit efflux pumps, and at the sight of the promising inhibitory results obtained, we wanted to explore whether the compounds were able to synergistically enhance the activity of commercial antibiotics against multidrug-resistant bacterial strains.

### 3.3. Quorum Sensing (QS) Inhibition and Anti-Biofilm Assay

Inhibition of bacterial cell to cell communication finds its application in the prevention and spreading of bacterial infections. The communication is used by bacteria to sense their count, and in specific breakpoints, they switch their behavior and start to produce biofilm, thus regulating their virulence and metabolism [28]. Nowadays, the quorum-sensing modulators offer new tools in the fight against bacterial resistance and in the diagnosis of the disease, and also act as novel antimicrobial agents. Quorum sensing is based on three types of molecules: homoserine lactones, peptides, and boron structures. AI-1 communication is based on homoserine lactones and is provided by LuxI protein, which is responsible for AI production, and LuxR protein, which becomes activated by AI [29]. AI-2 communication is based on boron structures, which are produced by LuxS, and recognized by the sensor kinase. Usually, the communication of Gram-negative bacteria is due to the homoserine lactones, whereas Gram-positive bacteria use peptides as AI-1 type of molecules. AI-2 molecules are more universal and serve for communication in both Gram-positive and Gram-negative bacteria. While the homoserine lactones can diffuse freely across the cell membrane, peptide autoinducers usually require special transport mechanisms. These transport mechanisms are generally provided by ABC transporters (ATP-binding cassette), which are similar to those used by mammalian cells as efflux pumps. The inhibitors of the bacterial communication based on peptides could therefore find an application in the inhibition of the related-mammalian ABC transporters, whose overproduction is responsible for, e.g., chemotherapeutic-resistant cancer or drug-resistant epilepsy [30].

On the basis of our results, several compounds appear promising for their use as communication inhibitors. Compound **K2** inhibited both types of communication with a significant selectivity to inhibit communication rather than growth of bacteria. This selectivity is favorable for non-pathogenic (symbiotic) bacteria that constitute the human microflora. The ketone-selenoester **K1** was evaluated as the most promising compound inhibiting AI-1-based communication, followed by **N2**, which was the only cyano-selenocompound that was capable of inhibiting this AI-1-based communication. Both **K1** and **N2** were able to inhibit the communication at concentrations as low as 0.25 and 0.34 μM, respectively. Furthermore, both the ketone-selenoester **K2** and the cyano-selenoester **N2** share a 2-fluorophenyl moiety bound to the selenoester, which seems important for this inhibition of AI-1 communication. Interestingly, the substitution with fluorine atoms in the absence of other substituents was profitable for the activity, as the fourth most active compound was the one with a 2,4,5-trifluoro substitution. Alternatively, the activity of the unsubstituted derivative **K1** (the most active inhibitor) may not be related to the lack of substitution because its nitrile equivalent (**N1**) is devoid of activity. The communication of *P. aeruginosa* is usually based on homoserine lactones (AI-1); therefore, its adhesion should be dominantly inhibited by the same compounds inhibiting AI-1-based communication of *V. campbellii* (strain BAA 1118). Compounds **K1**, **K2**, and **K8** showed QS selectivity indexes higher than 10, and they were also the most active inhibitors of the adhesion of *P. aeruginosa* in the anti-biofilm assay.

Otherwise, the cyano-selenocompounds were more effective in the inhibition of AI-2-based communication—**N3** and **N7** were the most effective compounds among the others, with **N3** being capable of exerting its inhibition at a concentration as low as 60 nM. Intriguingly, they had a quite different substitution at the phenyl ring than the compounds active in AI-1—a bulky bromine atom (**N3**) or a more bulky di-substitution with trifluoromethyl groups (**N7**). Furthermore, the two more potent inhibitors among the ketone-selenoesters also included bulky substituents—trifluoromethyl (**K4**) or *tert*-butyl (**K7**) derivatives that support this observation. Compound **K2**, with a fluorine atom, was also active, but with a selectivity index (SI) of 14.7, significantly lower than the ones of **N3**, **N7**, **K4**, and **K7**: 37.6, 30.8, 23.0, and 28.5, respectively.

Quorum sensing of Gram-positive bacteria is usually based on peptide molecules, which are not typical of *Vibrio* communication; therefore, these results could not be correlated. However, many of tested compounds showed a significant inhibition of *S. aureus* adhesion; thus, the Se-compounds should be investigated more in depth to determine their ability to modulate the activity of ABC transporters. Autoinducers-2 are commonly used by many Gram-positive and Gram-negative bacteria. For example, *S. aureus*, bacteria belonging to the *Enterobacteriaceae* family or to the genus *Bacillus*, use the ABC transporters as a part of their communication [31]. However, in the AI-2 system, these transporters are used for uptake of communication molecules [32]. This universal system of communication spreading in both Gram-positive and Gram-negative bacteria was significantly inhibited by compounds **K2**, **K4**, **K7**, **N3**, **N6**, and **N7**.

Regarding the anti-biofilm assay, all compounds were able to prevent the biofilm adhesion in the two bacterial strains evaluated (*S. aureus* ATCC 25923 and *P. aeruginosa* CCM 3955) at concentrations below 4 μM. Two of them (**K8** and **N3**) exerted this inhibition of the *P. aeruginosa* biofilm at nanomolar range: 0.86 μM and 0.92 μM, respectively. Seven additional compounds exerted this effect at concentrations from 1 to 2 μM in *P. aeruginosa*, whereas eight showed this range of activity against *S. aureus*. A tendency can be observed—the compounds monosubstituted with halogens (**K1**–**K3**, **N1**–**N3**), with the exception of **N1** and **N2**, tended to have anti-adhesion activity at concentrations below 2 μM. In the anti-biofilm evaluation, all compounds were disruptors of existing biofilms at concentrations below 25 μM in *P. aeruginosa*, and below 50 μM in *S. aureus*. Out of them, **K2**, **K3**, **K5**, and **K8** disrupted the biofilm at concentrations below 10 μM in *P. aeruginosa*, and **K8** in *S. aureus*. In this case, no SARs could be extracted, and besides this, the ketone-selenoesters resulted in being more potent disruptors than the cyano-selenoesters; moreover, the compounds were more effective against *P. aeruginosa* biofilms than against those of *S. aureus*.

## 4. Materials and Methods

### 4.1. Compounds

The 15 selenoesters evaluated in this work were previously synthesized and evaluated as described at the patent application EP17382693 [33]. Briefly, a selenation of an acyl chloride was initially performed in aqueous media, being the selenating agent, and sodium hydrogen selenide was prepared in situ by reduction of metallic selenium with sodium borohydride. Later, the intermediate generated, with no purification (one-pot synthesis), reacted with the adequate alkyl halide to render the desired selenoester. When necessary for not being commercially available, the acyl chloride was synthesized by the chlorination of the corresponding carboxylic acid using thionyl chloride.

Before each biological assay, the stock solution of selenoesters (10 mM) was prepared in dimethyl sulfoxide (DMSO).

### 4.2. Reagents and Media

DMSO (Sigma-Aldrich, St Louis, MO, USA), phosphate-buffered saline (PBS; pH 7.4), Mueller–Hinton (MH) broth, autoinducer bioassay (AB-A) medium, resazurin sodium salt (Sigma-Aldrich), tryptic soy broth (TSB), tryptic soy agar (TSA), brain heart infusion (BHI), Luria–Bertani broth (LBB), Luria–Bertani agar (LBA), reserpine, CCCP (carbonyl cyanide 3-chlorophenylhydrazone).

### 4.3. Bacterial Strains

As Gram-positive strains, *Staphylococcus aureus* American Type Culture Collection (ATCC) 25923 strain was used as methicillin-susceptible reference and biofilm-producing strain; the clinical isolate *S. aureus* MRSA 272123 and the methicillin and oxacillin-resistant *S. aureus* MRSA ATCC 43300 strains were investigated in the study.

As Gram-negative strains, the biofilm-producing *Pseudomonas aeruginosa* CCM 3955/ATCC 27853, multidrug-resistant *P. aeruginosa* NEM 986 strain, the wild-type *Salmonella enterica* serovar Typhimurium SL1344 (SE01) expressing the AcrAB-TolC pump system and its *acrB* gene-inactivated mutant *S. Typhimurium* SL1344 strain (SE02), *acrA* gene-inactivated mutant *S. Typhimurium* SL1344 (SE03), and *tolC* gene-inactivated mutant *S.* Typhimurium SL1344 strain (SE39) were used in the study. In terms of QS tests, the Gram-negative *Vibrio campbellii* ATCC BAA-1118 and ATCC BAA-1119 strains were applied. Microorganisms were obtained from the Czech Collection of Microorganisms (CCM, Masaryk University, Czech Republic) and the Collection of Laboratory of Medical Microbiology (NEM, Czech Laboratory, Ltd., Prague, Czech Republic).

### 4.4. Determination of Minimum Inhibitory Concentrations (MIC) by Microdilution Method

The minimum inhibitory concentrations (MICs) of ketone- and cyano-selenoesters were obtained according to the Clinical and Laboratory Standard Institute guidelines (CLSI) [34]. The MIC values of the compounds were established by visual inspection. The solvent DMSO did not exert any antibacterial activity. The MIC determination was performed in 4 parallels for each compound and strain, respectively.

### 4.5. Real-Time Ethidium Bromide Accumulation Assay

The efflux pump inhibiting activity of Se-compounds was tested on *S. aureus* ATCC 25923 and *S. aureus* MRSA ATCC 43300 strains by real-time fluorimetry monitoring the intracellular accumulation of the efflux pump substrate EB using a CLARIOstar Plus plate reader (BMG Labtech, Ortenberg, Germany). Reserpine (RES) was applied at 25 µM as a positive control; the solvent DMSO was applied at 1 v/v %. The bacterial strains were cultured at 37 °C in a shaking incubator until they reached an optical density (OD) of 0.6 at 600 nm. The cells were then washed with phosphate-buffered saline (PBS; pH 7.4) and centrifuged at 13,000× *g* for 2 min; the pellet was re-suspended in PBS. The Se-compounds were applied at one-half MIC concentration to PBS supplemented with a non-toxic concentration of EB (2 µg/mL). Then, the solutions were pipetted into a 96-well black microtiter plate (Greiner Bio-One Hungary Kft, Hungary), and 50 μL of bacterial suspension (OD_600_ 0.6) was pipetted to the wells. Then, the plates were inserted into the CLARIOstar plate reader, and the fluorescence was recorded at excitation and emission wavelengths of 530 nm and 600 nm, respectively, every minute for 1 hour. From the real-time data, the relative fluorescence index (RFI) of the last time point (minute 60) of the EB accumulation assay was calculated according to the subsequent equation:

RFI = (RF_treated_ − RF_untreated_)/RF_untreated_

where RF_treated_ is the relative fluorescence (RF) at the last time point of EB retention curve in the presence of an inhibitor, and RF_untreated_ is the RF at the last time point of the EB retention curve of the untreated control having the solvent control (DMSO) [24]. The RFI values were analyzed by *t*-test, and statistical significance was defined as *p* < 0.05.

### 4.6. Assay for Quorum Sensing (QS) Inhibition

Anti-QS activity was monitored by two commercial strains of *V. campbellii* (ATCC BAA-1118 and ATCC BAA-1119). The first one responds by bioluminescence to AI-1 inducer, the second one to AI-2 inducer [35]. The effect of compounds on the luminescence generation was evaluated as described previously. Briefly, the overnight culture of strains was diluted to 5 × 10^5^ CFU/mL (colony-forming units per milliliter) in Autoinducer Bioassay medium ((NaCl (17.5 g/L), MgSO4 (12.3 g/L), casamino acids (2 g/L), 10 mM potassium phosphate (pH 7.0), 1 mM L-arginine, and glycerol (10 mL/L of each)) and split into 96-well plates. After adding the compounds and their twofold serial dilutions, we incubated the plate for 8 h at 30 °C with continuous shaking. Then, luminescence was recorded for 16 h using a microplate reader (SpectraMax i3 Multi-Mode Detection Platform, Molecular Devices, San Jose, CA, USA) set up at 30 °C, integration time of 10,000 ms, and shaking for 60 s prior to measurement. The EC_50_ of compounds was determined on the basis of the sum of luminescence. After that, the viability of culture was determined by resazurin assay and the IC_50_ of compounds was calculated. The compounds were compared on the basis of EC_50_ (the concentration that halves the cell communication) and IC_50_ (viability). The EC_50_ and IC_50_ were calculated by using GraphPad Prism software version 5.00 for Windows with nonlinear regression curve fit (GraphPad Software, San Diego, CA, USA; www.graphpad.com).

### 4.7. Anti-Biofilm Activity

#### 4.7.1. Inhibition of Biofilm Formation

The effect of the Se-compounds on biofilm formation was investigated on *S. aureus* ATCC 25923 and *P. aeruginosa* CCM 3955 (ATCC 27853). The experiment was carried out in 96-well microplates [36]. The overnight bacterial was diluted in brain heart infusion (BHI) broth to achieve the optical density of 0.5 McFarland, and the suspension was distributed into 96-well plates in 100 μL aliquots per well. The Se-compounds were pipetted to the cells in a concentration range of 100 μM to 3.125 μM. The plate was kept for 24 h at 37 °C. Then, the viability of adherent cells was determined immediately by resazurin assay. The medium was discarded, the samples were washed 3 times by phosphate-buffered saline (PBS), and 100 µL of resazurin in PBS (0.03 mg/L) was added to the wells [37]. The viability was measured by recording the fluorescence (560/590 nm, ex./em.) by the SpectraMax i3x Multi-Mode Detection Platform (Molecular Devices, San Jose, CA, USA). The assays were performed in four parallels. The relative viability was evaluated as a percentage according to the formula:

$$\mathrm{RA}\;\left[\%\right]=100\frac{\mathrm{sample}\;\mathrm{fluorescence}\;–\;\mathrm{average}\;\mathrm{fluorescence}\;\mathrm{of}\;\mathrm{NC}}{\mathrm{average}\;\mathrm{fluorescence}\;\mathrm{of}\;\mathrm{PC}\;-\;\mathrm{average}\;\mathrm{fluorescence}\;\mathrm{of}\;\mathrm{NC}}$$

where RA is relative activity in percentage, PC is positive control (untreated biofilm), and NC is negative control (resazurin incubated without bacterial cells).

The IC_50_ values were calculated using the online tool freely provided by AAT Bioquest–IC50 Calculator.

#### 4.7.2. Disruption of Mature Biofilm

The activity of Se-compounds to damage mature biofilms formed by *S. aureus* ATCC 25923 or *P. aeruginosa* CCM 3955 (ATCC 27853) was investigated by resazurin assay [38]. The assay was carried out in 96-well plates. The overnight bacterial cultures were diluted in BHI broth to the optical density of 0.5 McFarland and pipetted in 100 µL aliquots into the wells. After 24 h of incubation at 37 °C, the medium was discarded, and fresh BHI broth containing Se-compounds was measured to the wells. After 24 h of incubation, the medium was discarded, the wells were washed 3 times by PBS (pH 7.4), and 100 µL of resazurin in PBS (0.03 mg/L) was measured to the samples. The viability was recorded by measuring fluorescence (560/590 nm, ex./em.) using the SpectraMax i3x Multi-Mode Detection Platform (Molecular Devices, San Jose, CA, USA). The assays were performed in 4 parallels. The IC_50_ values were calculated using the online tool freely provided by AAT Bioquest–IC50 Calculator.

## 5. Conclusions

This work describes the biological evaluation of 15 novel selenoesters as antibacterials that have a phenyl ring, with different substituents linked to the carbonyl and a functionalized alkyl chain linked to the selenium atom. Eight selenoesters (**K1**–**K8**) contain a ketone group in this chain, whereas the seven remaining (**N1**–**N7**) are functionalized by a cyano group. The ketone-selenoesters exerted a potent antibacterial activity against the three strains of *S. aureus* considered herein (one sensitive and two MRSA), higher than that observed for the cyano-selenoesters. Seven of the ketone derivatives showed submicromolar MIC values on *S. aureus* MRSA 272123. The antibacterial activity seemed to be reduced by the inclusion of bulky substituents. Regarding the inhibition of efflux pumps, compound **N4** was a more potent inhibitor than the reference reserpine in *S. aureus* MRSA 43300, and **K7** was a more potent inhibitor than the reference CCCP in *S. Typhimurium* SE39 Δ*tolC*. Furthermore, the substitution with *tert*-butyl or trifluoromethyl groups seemed to enhance the inhibition of efflux pumps. Different compounds inhibited selectively the two main types of quorum sensing (QS)—**K1**, **K2**, **K8**, and **N2** inhibited the AI-1 communication, whereas **K2**, **K4**, **K7**, **N3**, and **N7** inhibited the AI-2 communication. Generally, ketone-selenoesters were better inhibitors of AI-1 and cyano-selenoesters were better inhibitors of AI-2. Finally, all compounds were able to prevent biofilm formation at concentrations below 4 μM in both *S. aureus* ATCC 25923 and *P. aeruginosa* CCM 3955. At the same time, all compounds disrupted biofilms produced by *S. aureus* at concentrations below 50 μM, and *P. aeruginosa* biofilms at concentrations below 25 μM. All these observations highlight the promising antibacterial, efflux pump inhibitory, quorum sensing inhibitory, and anti-biofilm activity of these novel ketone- and cyano-selenocompounds.

## Figures and Tables

**Table 1:**
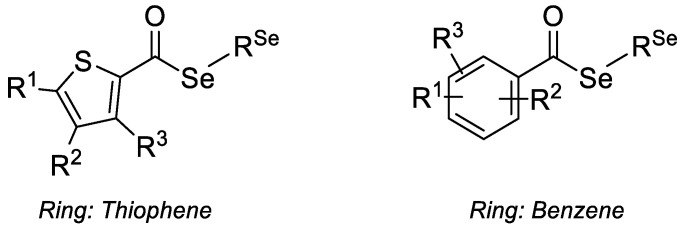
Ketone- and cyano-selenoesters evaluated. Cpds. = Compounds.

Cpds.	R^Se^	R^1^	R^2^	R^3^	Ring	Cpds.	R^Se^	R^1^	R^2^	R^3^	Ring
**K1**	-CH_2_COCH_3_	-H	-H	-H	Thiophene	**N1**	-CH_2_CN	-H	-H	-H	Thiophene
**K2**	-CH_2_COCH_3_	2-F	-H	-H	Benzene	**N2**	-CH_2_CN	3-F	-H	-H	Benzene
**K3**	-CH_2_COCH_3_	4-Br	-H	-H	Benzene	**N3**	-CH_2_CN	4-Br	-H	-H	Benzene
**K4**	-CH_2_COCH_3_	2-CF_3_	-H	-H	Benzene	**N4**	-CH_2_CN	2-CF_3_	-H	-H	Benzene
**K5**	-CH_2_COCH_3_	3-CF_3_	-H	-H	Benzene	**N5**	-CH_2_CN	3-CF_3_	-H	-H	Benzene
**K6**	-CH_2_COCH_3_	3-Cl	4-F	-H	Benzene	**N6**	-CH_2_CN	3-Cl	4-F	-H	Benzene
**K7**	-CH_2_COCH_3_	4-C(CH)_3_	-H	-H	Benzene	**N7**	-CH_2_CN	3-CF_3_	5-CF_3_	-H	Benzene
**K8**	-CH_2_COCH_3_	2-F	4-F	5-F	Benzene						

**Table 2:** Antibacterial activity of selenoesters on Gram-positive and Gram-negative bacteria (*S. aureus* = *Staphylococcus aureus, S.* Typhimurium = *Salmonella enterica* serovar Typhimurium, *P. aeruginosa* = *Pseudomonas aeruginosa*).

MIC Determination (µM)									
Cpds.	*S. aureus* ATCC 25923	*S. aureus* MRSA ATCC 43300	*S. aureus* MRSA 272123	*S.* Typhimurium** SE01 Wild-Type	*S.* Typhimurium** SE02 Δ*acrB*	*S.* Typhimurium** SE03 Δ*acrA*	*S.* Typhimurium** SE39 Δ*tolC*	*P. aeruginosa* CCM 3955	*P. aeruginosa* NEM 986
**K1**	1.56	1.56	0.78	50	100	100	100	100	50
**K2**	1.56	3.125	0.78	>100	>100	>100	>100	100	50
**K3**	1.56	3.125	0.78	50	50	50	50	>100	>100
**K4**	3.125	3.125	1.56	>100	>100	>100	>100	100	50
**K5**	1.56	3.125	0.78	100	50	50	>100	100	50
**K6**	1.56	3.125	0.39	100	100	100	100	100	50
**K7**	1.56	1.56	0.39	50	>100	100	>100	100	100
**K8**	1.56	1.56	0.78	50	>100	100	100	100	50
**N1**	12.5	100	25	50	50	100	100	>100	>100
**N2**	12.5	100	50	50	100	100	100	>100	>100
**N3**	12.5	50	25	50	50	50	50	>100	>100
**N4**	12.5	100	50	100	100	100	100	>100	>100
**N5**	12.5	50	50	100	100	100	100	>100	>100
**N6**	12.5	50	25	100	50	100	100	>100	>100
**N7**	12.5	50	25	50	50	50	100	>100	>100

**Table 3:** Efflux pump inhibitory effects of selenoesters on *Staphylococcus aureus* and *S.* Typhimurium strains in terms of RFI (relative fluorescence index) values. Higher RFI values indicate more efficient efflux pump inhibition.

Relative Fluorescence Index (RFI)						
Cpds.	*S. aureus* MRSA ATCC 43300	*S.aureus* ATCC 25923	*S.* Typhimurium SE01 wild-type	*S.* Typhimurium SE02 Δ*acrB*	*S.* Typhimurium SE03 Δ*acrA*	*S.* Typhimurium SE39 Δ*tolC*
**K1**	−0.02	1.19	0.17	0.20	0.29	0.31
**K2**	−0.04	1.12	0.18	0.21	0.30	0.42
**K3**	−0.10	1.17	0.60	0.27	0.24	0.44
**K4**	−0.09	1.02	0.19	0.35	0.68	0.95
**K5**	−0.08	1.17	0.41	0.11	0.35	0.91
**K6**	−0.09	1.19	0.17	0.43	0.52	0.80
**K7**	−0.02	1.13	1.02	0.30	1.15	1.67
**K8**	−0.04	1.10	0.08	0.31	0.28	0.70
**N1**	0.05	1.40	0.04	0.10	0.37	0.84
**N2**	−0.02	1.31	−0.06	−0.05	0.16	0.19
**N3**	−0.05	1.49	0.003	0.03	0.38	0.24
**N4**	0.35	1.78	0.22	0.39	0.36	1.00
**N5**	−0.03	1.43	0.02	0.02	0.32	0.43
**N6**	−0.01	1.32	0.03	−0.03	0.45	0.38
**N7**	−0.06	0.28	0.003	0.14	0.32	1.11
**CCCP**	-	-	3.37	1.83	3.30	1.61
**RES**	0.30	5.5	-	-	-	-

CCCP: carbonyl cyanide m-chlorophenyl hydrazone; RES: reserpine.

**Table 4:** Anti-quorum sensing effects of selenocompounds on *Vibrio* strains.

Cpd.	*Vibrio campbellii* BAA 1118			*Vibrio campbellii* BAA 1119		
	IC_50_ (µM)	EC_50_ (µM)	SI	IC_50_ (µM)	EC_50_ (µM)	SI
**K1**	5.76 ± 0.07	0.22 ± 0.01	26.2	2.02 ± 0.15	0.71 ± 0.05	2.8
**K2**	4.38 ± 0.47	0.25 ± 0.03	17.5	3.23 ± 0.14	0.22 ± 0.02	14.7
**K3**	1.12 ± 0.02	0.17 ± 0.01	6.6	0.77 ± 0.07	0.23 ± 0.02	3.3
**K4**	33.18 ± 3.45	4.68 ± 0.32	7.1	6.66 ± 0.13	0.29 ± 0.05	23.0
**K5**	2.42 ± 0.29	1.35 ± 0.03	1.8	1.32 ± 0.10	0.45 ± 0.01	2.9
**K6**	3.28 ± 0.19	2.29 ± 0.02	1.4	0.97 ± 0.09	1.20 ± 0.00	0.8
**K7**	10.54 ± 0.19	1.77 ± 0.19	6.0	4.27 ± 0.23	0.15 ± 0.01	28.5
**K8**	1.23 ± 0.07	0.11 ± 0.01	11.2	1.39 ± 0.03	0.46 ± 0.03	3.0
**N1**	2.21 ± 0.19	1.45 ± 0.02	1.5	2.28 ± 0.12	0.26 ± 0.03	8.8
**N2**	7.36 ± 0.70	0.34 ± 0.04	21.6	2.40 ± 0.12	0.73 ± 0.02	5.1
**N3**	2.199 ± 0.16	0.34 ± 0.04	6.5	2.35 ± 0.03	<0.06	37.6
**N4**	2.52 ± 0.03	1.29 ± 0.04	2.0	6.41 ± 0.42	0.73 ± 0.02	8.8
**N5**	12.51 ± 0.05	>5	-	3.57 ± 0.08	>5	-
**N6**	1.37 ± 0.02	0.37 ± 0.05	3.7	2.28 ± 0.12	0.22 ± 0.00	10.4
**N7**	3.84 ± 0.15	1.44 ± 0.06	2.7	7.71 ± 0.10	0.25 ± 0.02	30.8

**Table 5:** Concentration of selenoesters halving (IC_50_) the adhesion and disrupting the biofilm of *S. aureus* ATCC 25923 and *P. aeruginosa* CCM 3955 strains.

Compounds	*Staphylococcus aureus* ATCC 25923		*Pseudomonas aeruginosa* CCM 3955	
	Anti-Adhesion (μM)	Anti-Biofilm (μM)	Anti-Adhesion (μM)	Anti-Biofilm (μM)
**K1**	1.84 ± 0.26	32.80 ± 3.25	1.15 ± 0.01	10.21 ± 0.48
**K2**	1.72 ± 0.17	28.08 ± 1.17	1.10 ± 0.11	8.78 ± 0.66
**K3**	1.39 ± 0.13	11.64 ± 0.99	1.14 ± 0.05	6.00 ± 0.74
**K4**	3.59 ± 0.48	28.70 ± 4.18	3.04 ± 0.33	21.85 ± 2.04
**K5**	2.84 ± 0.13	15.44 ± 0.42	1.51 ± 0.22	6.45 ± 0.30
**K6**	2.96 ± 0.16	12.87 ± 0.37	2.33 ± 0.25	14.29 ± 1.62
**K7**	3.08 ± 0.24	40.80 ± 3.12	2.16 ± 0.29	11.06 ± 1.92
**K8**	1.35 ± 0.16	9.22 ± 0.61	0.86 ± 0.09	6.98 ± 0.22
**N1**	2.46 ± 0.15	24.79 ± 2.65	1.78 ± 0.07	15.51 ± 1.65
**N2**	3.14 ± 0.12	48.08 ± 3.82	2.86 ± 0.17	18.06 ± 0.72
**N3**	1.19 ± 0.15	30.46 ± 2.72	0.92 ± 0.01	10.56 ± 0.95
**N4**	1.49 ± 0.08	28.91 ± 2.00	2.49 ± 0.43	13.48 ± 0.82
**N5**	3.01 ± 0.35	34.55 ± 3.00	3.40 ± 0.10	24.81 ± 2.12
**N6**	1.83 ± 0.15	21.75 ± 2.61	1.34 ± 0.08	13.46 ± 1.77
**N7**	1.99 ± 0.26	16.53 ± 0.76	1.81 ± 0.04	11.09 ± 0.82
